# Hip arthroscopy in Sweden: a nationwide registry study of socioeconomic characteristics, regional variation, and sick leave patterns

**DOI:** 10.2340/17453674.2026.46648

**Published:** 2026-09-23

**Authors:** Miriam G WADSTRÖM, Anders STÅLMAN, Tobias WÖRNER, Frida EEK, Nils P HAILER, Jesper KRAUS-SCHMITZ, Yasmin D HAILER

**Affiliations:** 1 Section of Orthopaedics, Department of Surgical Sciences, Uppsala University; 2 Department of Molecular Medicine and Surgery, Section of Sports Medicine, Karolinska Institute, Stockholm; 3 Department of Health Sciences, Lund University, Lund; 4 Department of Orthopaedics, Skåne University Hospital, Malmö, Sweden

## Abstract

**Background and purpose:**

Clinical outcomes are well described after hip arthroscopy (HA), but less is known about the demographic, socioeconomic, and work-related characteristics of individuals undergoing the procedure at a population level. We aimed to describe demographic, socioeconomic, and regional characteristics of individuals undergoing HA in Sweden based on nationwide registry data compared with an age- and sex-matched reference population, and to describe regional variation in the annual incidence of HA using nationwide registry data.

**Methods:**

We conducted a nationwide descriptive registry study including all individuals aged 18–65 years who underwent HA in Sweden between 2006 and 2018. Each individual was matched by sex and year of birth to 5 reference individuals from the general population. National registers provided information on demographics, region of residence, education, citizenship, employment status, income, and compensated sick leave.

**Results:**

The study included 4,633 individuals undergoing HA and 23,060 matched reference individuals. Mean age was 36 years, and 3,019/4,633 (65%) were men. Residence-based HA incidence was highest in metropolitan and nearby counties. Individuals undergoing HA more often had post-secondary education (1,874/4,633 [40%] vs 8,003/23,060 [35%]), employment before surgery (4,299/4,633 [93%] vs 19,267/23,060 [84%]), and high-income levels (1,996/4,633 [43%] vs 7,358/23,060 [32%]) than the reference population. Compensated sick leave was more common among individuals undergoing HA both before surgery (741/4,633 [16%] vs 1,614/23,060 [7%]) and during the year after surgery (1,436/4,633 [31%] vs 1,845/23,060 [8%]).

**Conclusion:**

Individuals undergoing HA were more often employed and belonged to higher-income groups than a matched reference population. HA incidence varied substantially between regions, and individuals undergoing HA demonstrated higher levels of compensated sick leave both before and after surgery.

Hip arthroscopy (HA) is primarily indicated for femoroacetabular impingement syndrome, including labral tears in otherwise healthy young to middle-aged adults [1-3]. Over the past 2 decades, the procedure has become more commonly accepted. Sharp increases in annual incidence through the late 2000s and early-to-mid 2010s have been reported in several countries [4-6]. In Sweden, the incidence of the procedure peaked after a rapid increase around 2013–2014, after which a subsequent decline was noted [7]. The decline likely reflects evolving surgical indications, centralization of expertise, more restricted selection of patients, and accumulated experience, rather than a decline in surgical capacities [3,7,8].

Across large series, femoroacetabular impingement syndrome patients are typically active males, between 20 and 40 years of age, with a cam and/or pincer morphology and limited or no radiographic osteoarthritis [1,5,6,9]. Clinical studies generally report improvements in pain and function after hip arthroscopy, and several studies have also examined return to work [10,11]. While clinical results are widely reported, population-level characteristics remain poorly described. Trials and cohort studies rarely capture socioeconomic data or work status, and few analyses evaluate sickness absence or employment-related patterns alongside patient-reported outcome measures (PROMs) [12-15]. Prior Swedish registry initiatives for hip arthroscopy patients describe demographics and enable assessment of functional outcomes with PROMs [3], but a nationwide descriptive evaluation, including socioeconomic markers, sickness-absence patterns, and regional variations in annual procedure incidence, has not been reported.

The aims of this nationwide registry study were primarily to describe demographic, socioeconomic, and regional characteristics of individuals undergoing hip arthroscopy in Sweden compared with an age- and sex-matched reference population, and to describe regional variation in the annual incidence of HA using nationwide registry data; and secondarily to describe employment status and compensated sick-leave patterns before and after surgery.

## Methods

### Study design

We conducted a nationwide descriptive registry study of individuals undergoing HA in Sweden between January 1, 2006 and December 31, 2018. For descriptive comparison, each individual undergoing HA was matched by sex and year of birth to 5 reference individuals from the general population. The manuscript was prepared according to the Strengthening the Reporting of Observational Studies in Epidemiology (STROBE) guidelines [16].

### Data sources

We linked Swedish population registers using the personal identity number. The National Patient Register (NPR) (inpatient since 1987; specialist outpatient since 2001) provided dates of care, operating-room procedures, and diagnoses (ICD-7–10; Swedish procedure codes). From Statistics Sweden (SCB), we used the Total Population Register (frame for sampling the reference individuals) and the Longitudinal Integration Database for Health Insurance and Labour Market Studies (LISA) for sociodemographic and socioeconomic variables. The Health, Sickness, Income and Work (HSIA) database supplied harmonized indicators of labor-market attachment and earnings. From the Swedish Social Insurance Agency’s MiDAS database, we obtained the number of compensated sickness-benefit days per calendar year.

### Identification of individuals undergoing HA

Individuals undergoing HA were identified using a previously published selection algorithm by Wörner et al., combining procedure codes from the NPR with additional data sources. The algorithm was developed to improve case ascertainment because reporting of HA procedures to the NPR was incomplete mainly in the private health care sector. The algorithm has not been formally validated against an independent reference standard [7].

### Eligibility criteria of individuals undergoing HA and reference individuals

Eligible individuals were aged 18–65 years at the index date (date of HA). The age restriction was applied to focus analyses on a working-age population in which employment status, income, and compensated sick leave are relevant measures.

For each HA individual, Statistics Sweden selected 5 reference individuals from the general population matched on sex and year of birth. Reference individuals were required to be alive and resident in Sweden during the index year and to have no recorded HA procedure before or during the index year.

### Variables

From the National Patient Register, we obtained information on index year, procedure codes, and treating hospital region. Statistics Sweden provided sociodemographic variables (age, sex, civil status, citizenship, and county of residence), socioeconomic variables (education level and year-specific income quintiles), and employment status.

Income was categorized into year-specific quintiles (Q1–Q5), with zero income retained as a separate category. To facilitate descriptive comparisons, income quintiles were additionally collapsed into low- (Q1–Q2), middle- (Q3), and high-income (Q4–Q5) groups.

Employment status was categorized as employed or not employed. The employed category included both employees and self-employed individuals, whereas the not-employed category included individuals without registered employment, such as students, individuals on parental leave, job seekers, and others outside the labor market.

Information on compensated sick leave was obtained from the Swedish Social Insurance Agency MiDAS database. In Sweden, sickness-benefit compensation may be granted to individuals with reduced work capacity due to disease or injury following assessment by the Swedish Social Insurance Agency. Eligibility is not restricted to employed individuals and may also apply to certain self-employed, unemployed, and student populations who fulfil eligibility criteria. The MiDAS register captures compensated sickness absence administered through the national social insurance system but does not include shorter employer-paid sickness absences during the first 14 days of sick leave. Compensated sick-leave days were summarized for 3 predefined periods: the year before surgery, the index year (year of surgery), and the year after surgery (more than 12 months after surgery).

### Statistics

Descriptive statistics were used in this study. Continuous variables are presented as means with standard deviations (SD) or medians with interquartile ranges (IQR), depending on distribution. Categorical variables are presented as counts and percentages.

To facilitate interpretation of between-group differences independent of sample size, standardized mean differences (SMDs) were calculated for selected baseline characteristics using the “tableone” package in R (R Foundation for Statistical Computing, Vienna, Austria). For categorical variables with more than 2 levels, an overall multivariate SMD was calculated.

Annual incidence rates of HA surgery between 2006 and 2018 were calculated using annual population data obtained from Statistics Sweden. Incidence rates were expressed as the number of surgeries per 100,000 inhabitants aged 18–65 years for each calendar year. The population at risk was approximated using the mean population size at the beginning and end of the study period. Regional incidence rates were calculated similarly for the residential region and expressed per 100,000 inhabitants. Exact 95% confidence intervals were estimated assuming a Poisson distribution and visualized using point estimates with error bars.

Income was categorized using year-specific quintiles to account for secular changes in wages and inflation during the study period.

Compensated sick leave was summarized in 3 complementary ways:

1. the proportion of individuals with any compensated sick leave (≥ 1 day),
2. the median (IQR) number of compensated days among individuals with any sick leave, and
3. the mean (SD) number of compensated days in the total study population, including individuals without sick leave.

Because compensated sick leave demonstrated a highly skewed and zero-inflated distribution, both prevalence measures and summary measures of duration are presented.

All analyses were performed using R version 4.3.2.

### Missing data and data quality

Missing categorical data were retained as separate “missing/unknown” categories because the extent of missingness was low, and the analyses were descriptive in nature. No imputation procedures were performed. This approach was considered appropriate according to current Acta Orthopaedica guidance for reporting and handling missing data in observational research [17]. Registry linkage was performed using the Swedish personal identity number. Internal consistency checks, including duplicate control and validation of age relative to index year, were performed before analysis.

### Ethics, data sharing plan, funding, use of AI, and disclosures

This study was approved by the Swedish Ethical Review Authority (Dnr 32355/2021, Dnr 2018/183, Dnr 2019-04514, 2017/1253-31, 2021-04180, 2024-01-22) and conducted in accordance with the Declaration of Helsinki. Data was pseudonymized by the register holders before delivery to the research team. The data underlying this study is available from Swedish national registers, but restrictions apply to their availability. Data may be obtained from the respective authorities, subject to ethical approval and data access agreements.

No external funding was received. ChatGPT (OpenAI) was used to assist with language editing and preparation of the response to reviewers. All scientific content and final manuscript revisions were reviewed and approved by the authors. The authors declare no competing interests. Complete disclosure of interest forms according to ICMJE are available on the article page, doi: 10.2340/17453674.2026.46648

## Results

### Study population

**Figure 1:**
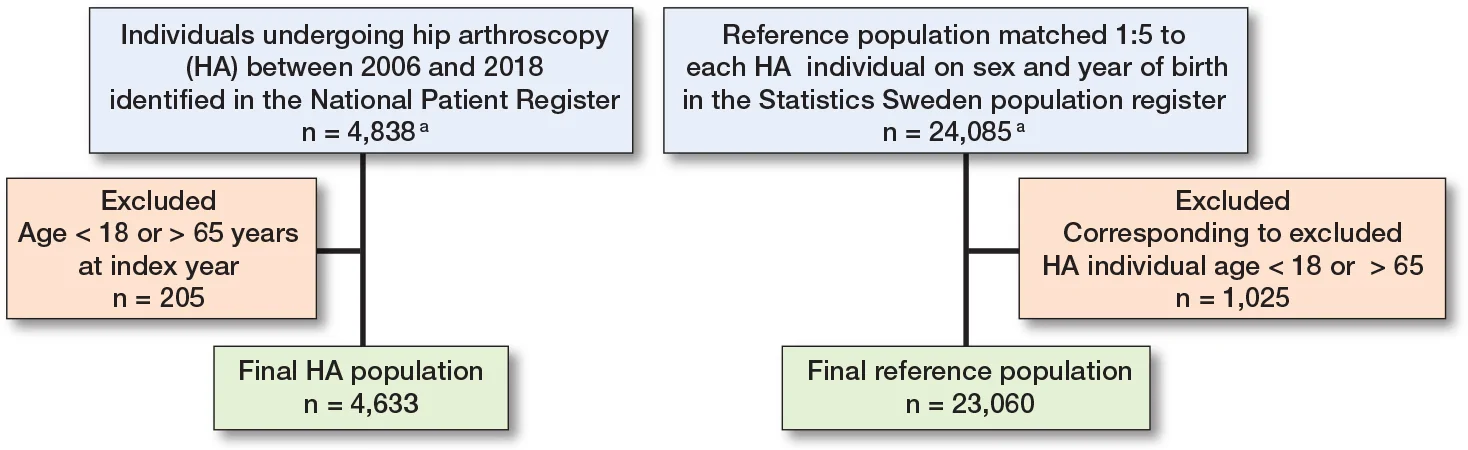
Eligibility and matching flow for the study population. ^**a**^ For 21 individuals undergoing HA, 5 eligible reference matches could not be identified, and these individuals, together with their partial matches, were excluded (105 reference individuals).

The study population consisted of 4,633 individuals undergoing HA and 23,060 matched reference individuals. For 21 individuals undergoing HA, 5 eligible reference matches could not be identified, and these individuals, together with their partial matches, were excluded (Figure 1).

### Demographics and socioeconomic profile

Mean age at index year was 36 years (SD 12), and 65% (3,019/4,633) were men among individuals undergoing HA. Post-secondary education was more common among individuals undergoing HA (1,874/4,633 [40%]) than in the reference population (8,003/23,060 [35%]), whereas lower educational attainment was less common (463/4,633 [10%] vs 3,550/23,060 [15%]). Swedish citizenship was more common among individuals undergoing HA (4,495/4,633 [97%]) than in the reference population (20,577/23,060 [89%]) (Table 1).

**Table 1:** Baseline characteristics of the study population. Values are n (%) unless otherwise stated

Characteristic	Reference population n = 23,060	Individuals undergoing HA n = 4,633	SMD ^a^
Sex (matched)			0.00
Female	8,050 (35)	1,614 (35)	
Male	15,010 (65)	3,019 (65)	
Age at index year, mean (SD)	36 (12)	36 (12)	0.00
Education level			0.22
Primary education (≤ 9 years)	3,550 (15)	463 (10)	
Upper secondary education	10,798 (47)	2,235 (48)	
Post-secondary education	8,003 (35)	1,874 (40)	
Missing/unknown	709 (3.1)	61 (1.3)	
Civil status			0.06
Unmarried	13,756 (60)	2,748 (59)	
Married/registered partner	7,361 (32)	1,561 (34)	
Divorced/widowed	1,865 (8.1)	312 (6.7)	
Missing/unknown	78 (0.3)	12 (0.3)	
Citizenship			0.35
Sweden	20,577 (89)	4,495 (97)	
Nordic countries (except Sweden)	242 (1.0)	47 (1.0)	
Europe (excluding Nordic countries)	896 (3.9)	53 (1.1)	
Outside Europe	1,216 (5.3)	26 (0.6)	
Missing/unknown/stateless	129 (0.6)	12 (0.3)	

a SMD = standardized mean difference. An absolute SMD > 0.10 is generally considered to indicate a meaningful between-group difference.

### Annual distribution

**Figure 2:**
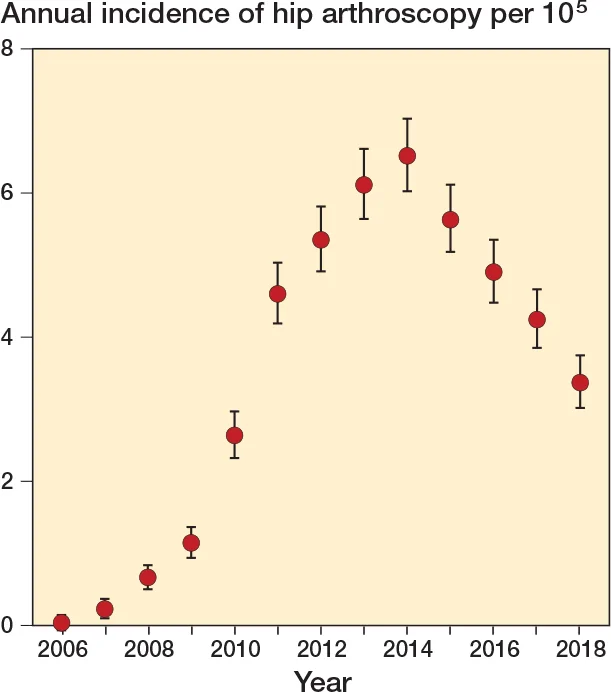
Annual incidence rate of patients who underwent hip arthroscopy per 100,000 per year with 95% confidence interval.

Annual incidence rates of HA increased from very few procedures in 2006 to a maximum of almost 7 per 100,000 inhabitants in 2014 (Figure 2). Thereafter, incidence rates declined steadily, reaching approximately 4 per 100,000 inhabitants in 2018.

### Regional distribution

**Figure 3:**
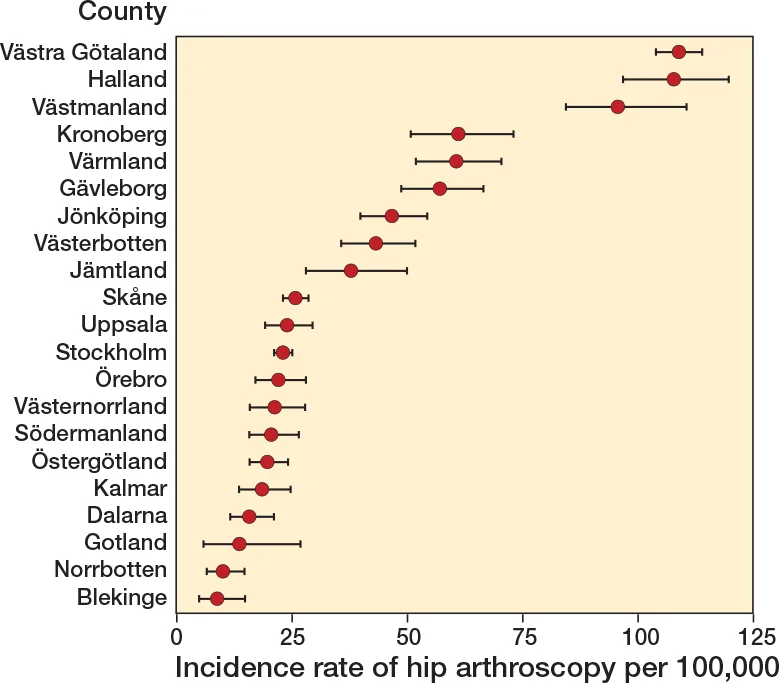
Residence-based incidence rates of hip arthroscopy by county in Sweden, 2006–2018 with 95% confidence interval.

Individuals undergoing HA were geographically concentrated in metropolitan and nearby regions. The largest proportions of individuals undergoing HA lived in Västra Götaland (1,842/4,633 [40%]) and Stockholm (509/4,633 [11%]). Residence-based incidence varied markedly between counties (Figure 3). The highest incidence was observed in Västra Götaland (approximately 110 per 100,000 inhabitants), followed by Halland (approximately 108 per 100,000) and Västmanland (approximately 95 per 100,000). Similarly, most procedures were performed in hospitals located in Västra Götaland (51%) and Stockholm (13%).

### Income distribution

Individuals undergoing HA were more often in higher-income categories than the reference population across all studied periods (Table 2). Before surgery, 1,996/4,633 (43%) individuals undergoing HA belonged to the high-income category compared with 7,358/23,060 (32%) in the reference population, whereas zero income was less common (328/4,633 [7%] vs 3,697/23,060 [16%]). Similar patterns were observed during the year of surgery and after surgery.

**Table 2:** Income categories before and after hip arthroscopy (HA). Values are count (%)

Period Group	Zero income	Low income (Q1–Q2)	Middle income (Q3)	High income (Q4–Q5)
Year before index year				
Reference	3,697 (16)	7,877 (34)	3,896 (17)	7,358 (32)
HA	328 (7.1)	1,493 (32)	783 (17)	1,996 (43)
Index year				
Reference	3,364 (15)	8,043 (35)	3,942 (17)	7,601 (33)
HA	306 (6.6)	1,517 (33)	834 (18)	1,948 (42)
Year after index year				
Reference	3,092 (14)	8,091 (35)	3,978 (18)	7,609 (33)
HA	277 (6.0)	1,515 (33)	822 (18)	1,983 (43)

Index year = year of hip arthroplasty.

### Employment status

Employment was more common among individuals undergoing HA than in the reference population across all studied periods (Table 3). Before surgery, 4,299 of 4,633 (93%) individuals undergoing HA were classified as employed compared with 19,267 of 23,060 (84%) in the reference population. Similar patterns were observed during the index year and the year following surgery.

**Table 3:** Employment status before and after hip arthroscopy (HA). Values are count (%)

Period/Group	Employed	Not employed	Missing
Year before index year			
Reference	19,267 (84)	3,561 (15)	232 (1.0)
HA	4,299 (93)	301 (6.5)	33 (0.7)
Index year			
Reference	19,716 (85)	3,236 (14)	108 (0.5)
HA	4,320 (93)	285 (6.2)	28 (0.6)
Year after index year			
Reference	19,807 (86)	2,963 (13)	290 (1.3)
HA	4,344 (94)	253 (5.5)	36 (0.8)

### Compensated sick leave

Compensated sick leave was more common among individuals undergoing HA than in the reference population across all studied periods (Table 4). Before surgery, 741/4,633 (16%) individuals undergoing HA had at least 1 compensated sick-leave day compared with 1,614/23,060 (7%) in the reference population. During the index year, the corresponding proportions were 2,687/4,633 (58%) and 1,845/23,060 (8%), respectively, and during the year following surgery 1,436/4,633 (31%) and 1,845/23,060 (8%).

**Table 4:** Compensated sick leave before and after hip arthroscopy (HA)

Period/Group	Any compensated sick leave, n (%)	Compensated days among affected, median (IQR) ^a^	Compensated days in total population, mean (SD)
Year before index year			
Reference	1,614 (7.0)	35 (12–97)	6 (33)
HA	741 (16)	51 (16–121)	14 (49)
Index year			
Reference	1,845 (8.0)	40 (14–117)	7 (34)
HA	2,687 (58)	50 (25–95)	45 (72)
Year after index year			
Reference	1,845 (8.0)	43 (14–121)	7 (38)
HA	1,436 (31)	51 (20–112)	26 (64)

a Compensated sick leave was measured as net compensated days registered in the Swedish Social Insurance Agency database. Median days are presented among individuals with at least 1 compensated sick-leave day.

Among individuals with any compensated sick leave, median numbers of compensated days were consistently higher among individuals undergoing HA. Mean numbers of compensated sick-leave days in the total study population, including individuals without sick leave, were also higher among individuals undergoing HA across all periods.

## Discussion

We aimed to describe the demographic, socioeconomic, and regional characteristics of individuals undergoing hip arthroscopy (HA) in Sweden, including regional variation in HA incidence and employment and sick-leave patterns, and showed that individuals undergoing HA in Sweden more often belonged to higher-income and employed groups compared with a matched reference population. HA incidence varied substantially between regions and was highest in metropolitan counties. Individuals undergoing HA also had higher levels of compensated sick leave both before and after surgery than the reference population.

### Socioeconomic patterns

Individuals undergoing HA more often had post-secondary education, employment, and higher income levels than the reference population. Several factors may contribute to these observed patterns. Femoroacetabular impingement syndrome is commonly diagnosed in physically active individuals [9], and participation in organized sports and recreational physical activity is known to vary across socioeconomic groups. Differences in healthcare-seeking behavior, occupational demands, referral pathways, geographic proximity to specialized centers, and access to private healthcare insurance may also influence which individuals ultimately undergo HA [14,18,19]. In addition, data from Statistics Sweden shows that average income levels are generally higher in Sweden’s metropolitan regions [20], where most HA procedures were performed. However, these regional income differences should be regarded as a descriptive population characteristic rather than an explanatory factor for access to surgery. Sweden has a tax-funded healthcare system with universal coverage, meaning that eligibility for specialist assessment and surgical treatment is not dependent on an individual’s financial resources or ability to pay.

Because the present study was descriptive and lacked information on symptoms, activity level, sports participation, disease severity, and referral indications, the findings should not be interpreted as evidence of inequitable access to HA. Rather, the results demonstrate socioeconomic differences among individuals undergoing the procedure and highlight the need for further research into factors influencing access, referral, and treatment selection.

### Regional variation

Marked regional variation in HA incidence was observed, with the highest residence-based incidence in metropolitan counties and a strong concentration of procedures to a limited number of treating regions. This likely reflects centralization of surgical expertise and referral patterns during the study period. HA is technically demanding and has increasingly been concentrated to specialized high-volume centers, which may contribute to regional differences in procedure utilization [3,7,8]. Unlike average income, employment rates do not consistently follow a metropolitan pattern in Sweden according to Statistics Sweden, suggesting that the observed differences in employment are unlikely to be explained solely by the regional concentration of HA procedures.

Several mechanisms may contribute to the observed regional pattern. Individuals living close to centers where HA is performed may have shorter travel distances, greater local awareness of the procedure among referring clinicians, and more established referral pathways. Conversely, patients living farther from specialized centers may face practical barriers related to travel, time away from work, and fragmented referral routes. Regional variation in surgery rates may also reflect differences in local diagnostic practices, treatment traditions, and physician preference, mechanisms that have been described more broadly for regional variation in surgical care [21]. Differences in access to private healthcare pathways may also have contributed, although this could not be assessed in the present data.

### Work-related findings

Individuals undergoing HA had higher levels of compensated sick leave both before and after surgery than the reference population. Increased sickness absence before surgery may reflect symptoms affecting physical activity, occupational demands, or healthcare utilization preceding surgical treatment. The marked increase during the index year likely reflects postoperative recovery and rehabilitation. It should be noted that compensated sick-leave days recorded in the Swedish MiDAS register reflect sickness-benefit compensation administered through the national social insurance system and may include both employed individuals and certain non-employed individuals who fulfil eligibility criteria. Consequently, compensated sick leave should be interpreted as a measure of work-capacity reduction and social insurance utilization rather than employment status alone.

However, interpretation of work-related patterns should be cautious, as the present study lacked information on occupation type, symptom severity, rehabilitation protocols, work demands, and return-to-work status. Previous studies have primarily focused on return-to-work outcomes after HA [10,13,22-24], whereas population-level patterns of compensated sick leave have been less studied. Our findings support the inclusion of work-related measures in future evaluations of HA populations.

### Strengths

The strengths of this study include the nationwide design, population-based registers with virtually complete follow-up, and linkage of healthcare and socioeconomic data at the individual level. The matched reference population enabled descriptive comparison of demographic and socioeconomic characteristics across a broad working-age population.

### Limitations

First, the study was descriptive and observational, and causal interpretations cannot be made. Second, registry data lacked information on symptom severity, radiographic findings, sports participation, physical activity level, occupation type, rehabilitation, and referral indications. These factors may influence both the likelihood of undergoing HA and work-related outcomes. Third, although national coverage was high, procedure identification partly relied on previously established algorithms because HA-specific coding practices varied during the early study period. Finally, compensated sick leave captures only physician-certified compensated absence and does not include shorter employer-paid sick leave periods.

### Conclusion

Individuals undergoing HA more often belonged to higher-income and employed groups compared with a matched reference population. HA incidence varied substantially between regions and procedures were concentrated to metropolitan centers. Individuals undergoing HA also demonstrated higher levels of compensated sick leave both before and after surgery. These findings provide nationwide descriptive data on demographic, socioeconomic, and work-related characteristics among individuals undergoing HA and may inform future research on healthcare utilization and treatment patterns.

## Notes

MW and YH conceived and designed the study. MW and YH performed the statistical analysis and interpretation of data. MW performed the statistical analyses and drafted the manuscript. AS, FE, JKS, and TW contributed to data collection and critically revised the manuscript. NH and YH contributed to the interpretation of the results and substantial revision of the manuscript. All authors reviewed and approved the final version of the manuscript.

Handling co-editors: Per Henrik Randsborg and Robin Christensen

*Acta* thanks Stein Atle Lie and Sverre Løken for help with peer review of this manuscript.

## References

[1] Frank J M, Harris J D, Erickson B J, Slikker III W, Bush-Joseph C A, Salata M J, et al. Prevalence of femoroacetabular impingement imaging findings in asymptomatic volunteers: a systematic review. Arthroscopy 2015; 31(6): 1199-204. doi: 10.1016/j.arthro.2014.11.042. 25636988

[2] Griffin D R, Dickenson E J, O’Donnell J, Agricola R, Awan T, Beck M, et al. The Warwick Agreement on femoroacetabular impingement syndrome (FAI syndrome): an international consensus statement. Br J Sports Med 2016; 50(19): 1169-76. doi: 10.1136/bjsports-2016-096743. 27629403

[3] Sansone M, Ahldén M, Jonasson P, Thomeé C, Swärd L, Baranto A, et al. A Swedish hip arthroscopy registry: demographics and development. Knee Surg Sports Traumatol Arthrosc 2014; 22(4): 774-80. doi: 10.1007/s00167-014-2840-9. 24464406

[4] Maradit Kremers H, Schilz S R, Van Houten H K, Herrin J, Koenig K M, Bozic K J, et al. Trends in utilization and outcomes of hip arthroscopy in the United States between 2005 and 2013. J Arthroplasty 2017; 32(3): 750-5. doi: 10.1016/j.arth.2016.09.004. 27793498

[5] Nepple J J, Vigdorchik J M, Clohisy J C. What is the association between sports participation and the development of proximal femoral cam deformity? A systematic review and meta-analysis. Am J Sports Med 2015; 43(11): 2833-40. doi: 10.1177/0363546514563909. 25587186

[6] Palmer A J R, Malak T T, Broomfield J, Holton J, Majkowski L, Thomas G E R, et al. Past and projected temporal trends in arthroscopic hip surgery in England between 2002 and 2013. BMJ Open Sport Exerc Med 2016; 2(1): e000082. doi: 10.1136/bmjsem-2015-000082. PMC5117047 27900161

[7] Wörner T, Eek F, Kraus-Schmitz J, Sansone M, Stålman A. Rapid decline of yearly number of hip arthroscopies in Sweden: a retrospective time series of 6,105 hip arthroscopies based on a national patient data register. Acta Orthop 2021; 92(5): 562-7. doi: 10.1080/17453674.2021.1928396. PMC8519534 34018896

[8] McCulloch P, Altman D G, Campbell W B, Flum D R, Glasziou P, Marshall J C, et al. No surgical innovation without evaluation: the IDEAL recommendations. Lancet 2009; 374(9695): 1105-12. doi: 10.1016/S0140-6736(09)61116-8. 19782876

[9] Mascarenhas V V, Rego P, Dantas P, Morais F, McWilliams J, Collado D, et al. Imaging prevalence of femoroacetabular impingement in symptomatic patients, athletes, and asymptomatic individuals: a systematic review. Eur J Radiol 2016; 85(1): 73-95. doi: 10.1016/j.ejrad.2015.10.016. 26724652

[10] Blaeser A M, Mojica E S, Mannino B J, Youm T. Return to work after primary hip arthroscopy: a systematic review and meta-analysis. Am J Sports Med 2023; 51(5): 1340-6. doi: 10.1177/03635465211064271. 35384746

[11] Ifabiyi M, Patel M, Cohen D, Simunovic N, Ayeni O R. Return-to-sport rates after hip arthroscopy for femoroacetabular impingement syndrome in flexibility sports athletes: a systematic review. Sports Health 2024; 16(6): 982-90. doi: 10.1177/19417381231217503. PMC11531010 38152899

[12] Kotlier J L, Fathi A, Kumaran P, Mayfield C K, Orringer M, Liu J N, et al. Demographic and socioeconomic patient data are rarely included in randomized controlled trials for femoral acetabular impingement and hip arthroscopy: a systematic review. Arthrosc Sports Med Rehabil 2024; 6(2): 100901. doi: 10.1016/j.asmr.2024.100901. PMC10878849 38379603

[13] Lee S, Cvetanovich G L, Mascarenhas R, Wuerz T H, Mather RC, Bush-Joseph C A, et al. Ability to return to work without restrictions in workers compensation patients undergoing hip arthroscopy. J Hip Preserv Surg 2016; 4(1): 30-8. doi: 10.1093/jhps/hnw037. PMC5467422 28630718

[14] Prabhavalkar O N, Carbone A D, Bruning R E, Domb E S, Maldonado D R. Effect of socioeconomic status on outcomes after hip arthroscopy: minimum 5-year follow-up. Am J Sports Med 2025; 53(14): 3347-55. doi: 10.1177/03635465251383908. 41272922

[15] Ramadanov N, Lettner J, Voss M, Prill R, Hable R, Dimitrov D, et al. Minimal clinically important differences in conservative treatment versus hip arthroscopy for femoroacetabular impingement syndrome: a frequentist meta-analysis of RCTs. Orthop Surg 2025; 17(9): 2514-28. doi: 10.1111/os.70097. PMC12404880 40616355

[16] von Elm E, Altman D G, Egger M, Pocock S J, Gøtzsche P C, Vandenbroucke J P, et al. The Strengthening the Reporting of Observational Studies in Epidemiology (STROBE) statement: guidelines for reporting observational studies. Lancet 2007; 370(9596): 1453-7. doi: 10.1016/S0140-6736(07)61602-X. 18064739

[17] Christensen R, Ranstam J, Overgaard S, Wagner P. Guidelines for a structured manuscript: statistical methods and reporting in biomedical research journals. Acta Orthop 2023; 94: 243-9. doi: 10.2340/17453674.2023.11656. PMC10176201 37170796

[18] Buerba R A, Dalton J, Sadhwani S, Schulz W, Atte A C, Vyas D. Hip arthroscopy utilization disparities and complications amongst ethnic groups. Inquiry 2024; 61: 00469580241282644. doi: 10.1177/00469580241282644. PMC11487505 39410760

[19] Sieurin L, Josephson M, Vingård E. Positive and negative consequences of sick leave for the individual, with special focus on part-time sick leave. Scand J Public Health 2009; 37(1): 50-6. doi: 10.1177/1403494808097171. 19141555

[20] Statistikmyndigheten SCB [Internet]. [cited 2026 Aug 1]. Inkomster för personer i Sverige. Available from: https://www.scb.se/hitta-statistik/sverige-i-siffror/utbildning-jobb-och-pengar/inkomster-for-personer/

[21] Birkmeyer J D, Reames B N, McCulloch P, Carr A J, Campbell W B, Wennberg J E. Understanding regional variation in the use of surgery. Lancet 2013; 382(9898): 1121–9. doi: 10.1016/S0140-6736(13)61215-5. PMC4211114 24075052

[22] Horner N, Chapman R S, Larson J, Hevesi M, Nho S J. Workers’ compensation patients undergoing hip arthroscopy for femoroacetabular impingement syndrome experience worse mid-term outcomes but similar return-to-work: a propensity-matched analysis at 5-year follow-up. Arthroscopy 2023; 39(11): 2293-9.e1. doi: 10.1016/j.arthro.2023.03.023. 37100215

[23] Cohn M R, Wichman D M, Newhouse A C, Mehta N, Fu M C, Chahla J, et al. High rate of full duty return to work after hip arthroscopy for femoroacetabular impingement syndrome in workers who are not on workers’ compensation. Am J Sports Med. 2021; 49(3): 729-36. doi: 10.1177/0363546520985517. 33534611

[24] Li Z I, Shankar D S, Garra S, Hughes A J, Triana J, Blaeser A M, et al. Return to work following hip arthroscopy for femoroacetabular impingement syndrome: a case series. Surgeries 2023; 4(3): 391-400. doi :10.3390/surgeries4030039.

